# Lymphatic filariasis in Fiji: progress towards elimination, 1997–2007

**DOI:** 10.1186/s41182-020-00245-4

**Published:** 2020-10-28

**Authors:** Rosie K. Manolas, Mike Kama, Merelesita Rainima-Qaniuci, Vinaisi D. Bechu, Samuela Tuibeqa, Mareta V. Winston, Nomeeta Ram, Flora Naqio, Kazuyo Ichimori, Corinne Capuano, Masayo Ozaki, Sung Hye Kim, Padmasiri Aratchige, Aalisha Sahukhan, Patricia M. Graves

**Affiliations:** 1 College of Public Health, Medical and Veterinary Sciences and JCU WHO Collaborating Centre for Vector-Borne and Neglected Tropical Diseases, College of Public Health, James Cook University, Cairns and Townsville, Queensland Australia; 2 Fiji Centre for Disease Control, Ministry of Health and Medical Services, Suva, Fiji; 3 WHO Office of Pacific Support, Suva, Fiji; 4 Nagasaki University, Nagasaki, Japan; 5 Department of Environmental Biology and Medical Parasitology, College of Medicine, Hanyang University, Seoul, Republic of Korea; 6 Castle Hill, NSW Australia

**Keywords:** Lymphatic filariasis, Fiji, Parasitic disease, Vector, Elimination, PacELF

## Abstract

**Background:**

Lymphatic filariasis (LF) is a major public health problem in the Pacific Region, including in Fiji. Through transmission by the mosquito vector *Aedes*, Fiji has suffered the burden of remaining endemic with LF despite efforts at elimination prior to 1999. In the year 1999, Fiji agreed to take part in the Pacific Programme for Elimination of LF (PacELF) and the Global Programme to Eliminate LF.

**Methods:**

This study reviewed and collated past data on LF in Fiji between 1997 and 2007. Sources included published papers as well as unpublished PacELF and WHO program meeting and survey reports. Records were held at Fiji’s Department of Health and Medical Services, James Cook University and the WHO office in Suva, Fiji.

**Results:**

Baseline surveys between 1997 and 2002 showed that Fiji was highly endemic for LF with an estimated 16.6% of the population antigen positive and 6.3% microfilaria positive at that time. Five rounds of annual mass drug administration (MDA) using albendazole and diethylcarbamazine commenced in 2002. Programmatic coverage reported was 58–70% per year, but an independent coverage survey in 2006 in Northern Division after the fifth MDA suggested that actual coverage may have been higher. Monitoring of the program consisted of antigen prevalence surveys in all ages with sentinel and spot check surveys carried out in 2002 (pre MDA), 2004, and 2005, together with knowledge, attitude, and practice surveys. The stop-MDA survey (C survey) in 2007 was a nationwide stratified cluster survey of all ages according to PacELF guidelines, designed to sample by administrative division to identify areas still needing MDA. The national antigen prevalence in 2007 was reduced by more than a third to 9.5%, ranging from 0.9% in Western Division to 15.4% in Eastern Division, while microfilaria prevalence was reduced by almost four-fifths to 1.4%. Having not reached the target threshold of 1% prevalence in all ages, Fiji wisely decided to continue MDA after 2007 but to move from nationwide implementation to four (later five) separate evaluation units with independent timelines using global guidelines, building on program experience to put more emphasis on increasing coverage through prioritized communication strategies, community participation, and morbidity alleviation.

**Conclusion:**

Fiji conducted nationwide MDA for LF annually between 2002 and 2006, monitored by extensive surveys of prevalence, knowledge, and coverage. From a high baseline prevalence in all divisions, large reductions in overall and age-specific prevalence were achieved, especially in the prevalence of microfilariae, but the threshold for stopping MDA was not reached. Fiji has a large rural and geographically widespread population, program management was not consistent over this period, and coverage achieved was likely not optimal in all areas. After learning from these many challenges and activities, Fiji was able to build on the progress achieved and the heterogeneity observed in prevalence to realign towards a more stratified and improved program after 2007. The information presented here will assist the country to progress towards validating elimination in subsequent years.

## Background

Lymphatic filariasis (LF) is a parasitic disease of humans, transmitted through a mosquito vector carrying the pathogenic worm. It can take 5–10 years for initial symptoms to occur [1]. The delayed onset of LF symptoms is one of the reasons that it has been able to reach endemic proportions in so many countries, as unsuspecting infected persons spread the parasite when a mosquito transfers it from one infected person to the next. Other reasons include lack of resources or prioritization of the disease and neglect of those suffering [2]. Although LF does not directly lead to death, it can cause severe chronic disability, including limb and scrotal swelling [3]. Three species of parasitic worms exist worldwide: *Wuchereria bancrofti*, *Brugia malayi*, *and B. timori*, which are transmitted via a number of mosquito genera including *Anopheles*, *Aedes*, *Culex*, and *Mansonia* [4].

### Global Programme to Eliminate LF

In 1997, the World Health Assembly resolved to eliminate LF as a public health problem [5] through use of mass drug administration (MDA) using a single annual dose of deworming drugs for a period of 4–6 years to reduce blood microfilariae (Mf) in LF-affected patients and block transmission. The program also aimed to reduce and prevent the morbidity and disability that occurs in chronic LF [6, 7], including reducing the number of acute dermatolymphangioadenitis attacks. By 2014, the GPELF had greatly reduced the estimated number of cases worldwide, but there were still an estimated 68 million persons affected by LF, with 36 million microfilaraemic persons, 19 million hydrocoele cases, and 17 million lymphedema cases [8].

### Pacific Programme to Eliminate LF

The Pacific Programme to Eliminate LF **(**PacELF) program was launched under the auspices of WHO, the Secretariat of the Pacific Community (SPC), and donor agencies in 1999 [9]. At that time, 16 of the 22 countries and territories in the Pacific Region (including Fiji) were classified as endemic by the criterion of LF population prevalence of > 1% in any part of the country [10].

Under PacELF guidelines, target dates were recommended for surveillance milestones to monitor program implementation and provide support as needed. The baseline survey before the start of MDA, also known as A survey, used rapid immunochromatographic tests (ICT) to determine antigen prevalence. During the MDA rounds, sentinel site surveys referred to as the B surveys, monitored program progress. After the fifth round of MDA, a nationwide antigen survey in all ages (the C survey) was scheduled to determine whether LF prevalence was low enough for the MDA to be stopped [9]. Once that milestone was reached, PacELF guidelines called for a D survey at least 2 years later in 5-year-old children to confirm interruption of transmission. The PacELF C and D surveys have now been replaced by current global guidelines which call for a stop-MDA survey and two further post-MDA transmission assessment surveys (TAS) in 6–7-year olds at 2–3-year intervals, before the program can be “validated” as having achieved elimination of LF as a public health problem [11, 12].

### The Fiji islands

**Fig. 1:**
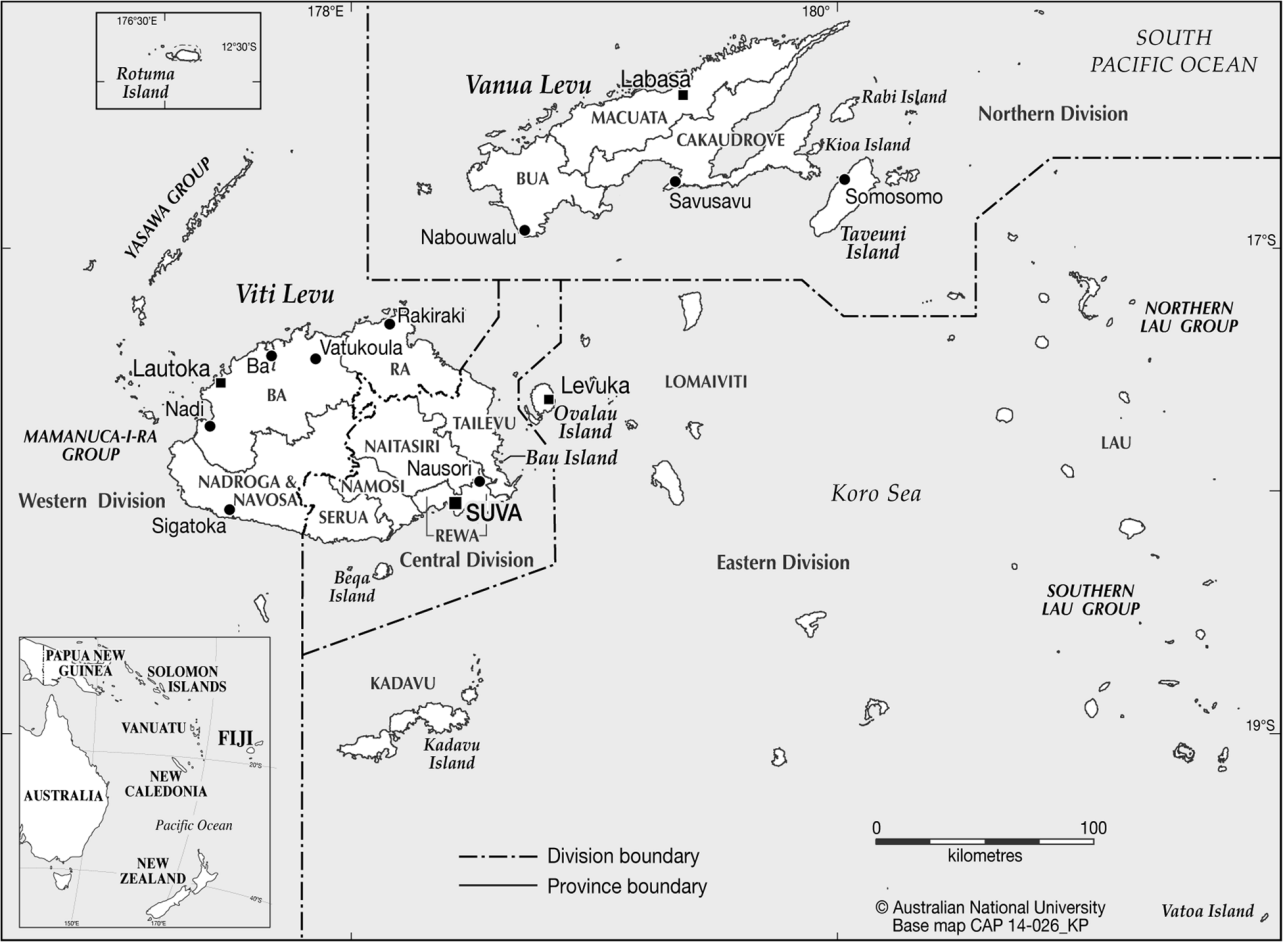
Map of Fiji with division and subdivision (province) boundaries. Source: Fraenkel (2015) Journal of Pacific History 2015 DOI: 10.1080/00223344.2015.1013598, with permission. Map produced by CartoGIS Services, ANU College of Asia and the Pacific, Australian National University (reproduced with permission)

Fiji is a group of more than 300 islands spanning 18,000 km^2^ of the Pacific Ocean (Fig. 1). There are four divisions and 15 subdivisions, also known as provinces. The capital is Suva, in the Central Division. Fiji was home to an estimated 837,271 people in 2007 which has grown to 884,887 by the 2017 census [13]. The population in 2007 was 56.8% ethnic Fijians (*iTaukei*), 37.5% Indian, 5.6% Chinese, 3.5% European, 1.8% other Pacific Islanders, 1.2% Rotuman, and 1.7% mixed or other. The median age in 2017 was 27.5 years and 55.9% of the population live in urban areas, an increase from 50.7% in 2007.

### Lymphatic filariasis in Fiji

In Fiji, *W. bancrofti* is transmitted predominantly by highly efficient *Aedes* vectors [9]. The known mosquito vector species are *Ae polynesiensis*, *Ae pseudoscutellaris*, *Ae horrescens*, *Ae rotumae*, *and Ae fijiensis*. Fiji has a history of being highly endemic for *W. bancrofti* LF, which was described there as early as 1876 [14, 15].

The first attempt to eliminate LF in Fiji was in 1944 through a mass vector control scheme with the aim of eliminating mosquito breeding sites, or through use of insecticides such as dichloro-diphenyltrichloroethane (DDT) [9]. In the late 1940s and early 1950s, there was experimental use of diethylcarbamazine (DEC) [16], but the first full scale MDA in Fiji started in 1958 [17] in a population of around 1500 people in Rewa district near Suva (see Fig. 1). An Mf prevalence of 12.2% (*N* = 1200) was found prior to the 1958 MDA, which consisted of 6 weekly doses of 6 mg/kg. The second course of 6 weekly doses was given 6 months later in early 1959, and 6 months after that the Mf prevalence in the study area was 2.7% (*N* = 1123). Subsequent testing in the years after MDA showed a rise in Mf prevalence, and in 1963, the Mf prevalence in the study area was 5.5% (*N* = 1430) [18].

Ministry of Health pre-MDA surveys conducted throughout Fiji starting in 1968 are summarized in the PacELF Way book [9]. Testing in 1969 on Taveuni and Koro islands revealed an Mf prevalence of 23% (*N* = 947), while in Vanua Levu, it was 13% (*N* = 3538). An MDA starting in 1969 in Fiji [19] once again attempted to control LF using 5 mg/kg of DEC weekly for 6 weeks, followed by treatment with the same dose on a monthly basis over 22 months. This MDA was carried out across the entirety of Fiji for a 2-year cycle in five stages and was completed in 1975, resulting in Mf prevalence in all treated areas decreasing to 1% or less [20, 21].

A mass Mf survey in 1983–1984 in the remote Fijian islands of Lau (*N* = 2329) and Rotuma (*N* = 1689) found that there was still high endemicity, since the prevalence of Mf was 7.9% and 21.2% respectively [15]. This apparent resurgence of LF led to the Filariasis Control Pilot Project in Fiji between 1984 and 1991 in three areas (Kadavu, Lomaiviti, and Rotuma). One area was administered DEC at 6 mg/kg annually for 5 years, the second was administered DEC at 5 mg/kg weekly for 6 weeks, then the same dose monthly for the following 22 months, and the third area did not have DEC administered for the initial 3-year period [15]. Results of the Filariasis Control Pilot Project saw the first two regions having marked reduction of Mf prevalence. Important research demonstrating the efficacy of LF annual single dose was carried out on Kadavu island during 1985–1990 [22]. Apart from some trials of DEC and ivermectin in Eastern Division in 1996 [9], no further MDA efforts were conducted until the start of PacELF in 1999.

Regarding morbidity, a survey carried out between 1991 and 1995 assessed 18,253 people (probably adults, but of unknown age) and found that 2733 were affected by advanced stage LF lymphedema and/or hydrocele (reported in [23]). Estimates based upon the above survey suggest the LF morbidity prevalence in adults at that time could have been as high as 17% [23]. However, the method or location of sampling in these surveys is not known, so these results should be considered of unknown accuracy; no further information on morbidity during the time period of this study is available.

A cross-cultural comparison of how LF affects Fiji’s population found that iTaukei had higher LF prevalence than Indo-Fijians. This may be because ethnicity influences the degree of exposure to mosquitoes, with indigenous Fijians being more likely to work in agricultural disciplines and live in more remote areas [24].

In 1999, Fiji recommitted to elimination of LF with the support of PacELF following the strategies described above. This paper reviews the activities and surveys conducted until 2007. During this period, there was 5 years of MDA, a vector control evaluation, knowledge-attitude-practice surveys, baseline and sentinel site surveys, a representative large C survey, and a MDA coverage survey [25, 26]. Fiji continued MDA after 2007, and the post-MDA surveillance period and TAS survey results will be reported separately.

## Methods

This data included in this paper were collected from published and unpublished reports and data from PacELF and Fiji Ministry of Health and Medical Services (MOHMS) databases relevant to PacELF in Fiji between the years of 1997 and 2007. Records of all sites surveyed (number tested and number positive by ICT or Mf blood slide) and the MDA coverage were extracted from PacELF databooks in 2003 and 2006 [25, 26] and the PacELF book [9]. Documents held at the JCU WHO Collaborating Centre PacELF Warehouse (jcu.edu.au/pacelf) were searched.

All data in this paper were collected and reported under PacELF or Fiji MOHMS supervision. Reports on events occurring in Fiji that noted obstacles, successes, and plan variations to the PacELF guidelines specifically for Fiji have been used to create an overview of Fiji’s PacELF progress. This paper will discuss Fiji’s PacELF journey, as well as the successes and challenges Fiji has undergone in efforts to eliminate LF during the years 1997 to 2007.

Antigen prevalence was the main method used to determine positive LF infection in the surveys reported. Microfilaria prevalence was also determined in some surveys using finger prick blood slides collected from those who were antigen positive, stained, and read according to WHO guidelines [11].

## Results

### Program overview

A timeline of activities is shown in Table 1, which outlines survey events and MDA implemented. Mapping (A) surveys using ICT antigen tests to determine baseline endemicity were underway in Fiji starting in 1997, and provided data for determining that MDA was needed everywhere in the country.

**Table 1:** Timeline of PacELF events, surveys, and MDA in Fiji 1997–2007

Year	Events	Surveys	MDA
1997		Baseline (A) surveys 1997–2001: Rotuma (N = 97)	
1998			
1999			
2000	National Plan of Action finalized. National Committee formed. National Policy developed. Nationwide education and awareness campaign on MDA and LF.	Baseline (A) survey 1997–2001 continued: Mapping survey (by ICT) to confirm endemic LF status in Fiji and the need for MDA: convenience sampling of 5893 people in > 45 sites across Fiji	
2001	July 2001—PacELF home office established at the Fiji Centre for Communicable Disease Control in Tamavua		
2002	Public health nurses trained; health promotion material distributed and campaign for MDA underway	Continued village surveys pre-MDA: convenience testing (by ICT and Mf) in 3214 persons of all ages in 48 villages. All positive cases were treated.	MDA1
2003	Full-time PacELF coordinator is established. Knowledge, attitudes, and practices (KAP) questionnaire administered by health professionals to MDA participants over 16.	Follow-up testing and treatment of positive cases.	MDA2
2004	MDA promotional material distribution.	Sentinel site (B) and spot check surveys: convenience testing (by ICT) in persons of all ages, in 14 villages; all positive cases treated.	MDA3
2005	Advocacy and awareness campaign managed by specially appointed awareness committee. Bednet study in Rewa district. Knowledge, attitudes, and practices (KAP) questionnaire administered by health professionals to MDA participants over 16. Commencement of radio and television MDA promotional broadcasts in Fijian, Hindi, and English.	Sentinel site (B) and spot check surveys: convenience testing (by ICT) in persons of all ages in 46 villages; all positive cases treated.	MDA4
2006	Global Alliance meeting in Fiji—Theme of “Global Elimination of LF: Successes and Challenges.” Program review including review of the MDA coverage results and communication strategy.	Follow-up: testing (by ICT) and treatment of positive cases. Coverage survey administered in Northern Division to assess MDA 5.	MDA5
2007	For the purpose of future LF elimination planning, identifying at-risk areas and presenting data, Fiji program is reorganized into 4 divisions—Eastern, Northern, Western, and Central.	Stop-MDA survey: ICT and Mf of all ages (C Survey) in 6745 people in 65 villages. Results > 1% positive in all but Western Division.	No MDA

In the second half of the year 2000, a draft National Plan of Action for LF elimination was finalized, a National Committee for LF Elimination in Fiji was established and a National Policy for the Filariasis campaign was created. December 2000 saw awareness workshops in all of Fiji’s division communities and memos sent to the public about further blood surveillance that commenced in January 2001 [9]. The PacELF plan of action for Fiji saw MDA as the primary intervention. It was to be carried out on all islands for the entire population, with the only participant exclusions being children under 24 months, pregnant women and people considered to be extremely sick (hospitalized, on dialysis or with cancer). Vector control appropriate to the environment was recommended in conjunction with MDA, and clinical management to relieve symptoms and prevent progression of advanced LF was to be performed where relevant.

### Mass drug administration

#### MDA treatment policy

The drugs DEC and albendazole were the official MDA treatments and were administered by age group based upon assumed weight and age [27] (Table 2).

**Table 2:** Dosage chart used in Fiji

Age group (years)	Albendazole dosage 400 mg, number of 400 mg tablets	DEC (target dose 6 mg/kg), number of 50 mg tablets
2–4	1	2
5–9	1	3
10–14	1	6
15–19	1	7
20–49	1	9
50 +	1	8

Exclusion criteria of MDA treatment:

- Sick individuals who are unable to take medications
- Children less than 2 years of age unable to take medication
- Pregnant women (however, postpartum lactating women will not be excluded)

#### MDA delivery strategies

The first MDA protocol involved door-to-door delivery of the MDA drugs albendazole and DEC. Initially, there was shortage of both human and financial resources for the 3–4-month period, especially in transporting persons to remote villages. In the second through to the fifth rounds of MDA, the protocol changed, so people were expected to go to a pick-up point over a certain weekend, where a health worker would provide the DEC and albendazole to be taken later at home. Prior to the weekend that the MDA was delivered, there was a mass MDA promotion campaign in urban regions and villages involving public figures and a range of media so people would be encouraged and prepared to take part in MDA [9]. Details of the usual MDA strategy are described in [27].

#### MDA coverage

Table 3 shows the percentage of population estimated to have taken part in the MDA program, as determined from programmatic records and the estimated census population.

**Table 3:** Programmatic MDA coverage in Fiji by year

	2002, MDA1	2003, MDA2	2004, MDA3	2005, MDA4	2006, MDA5
Population	841,500	841,500	841,500	841,500	849,361
Registered population of all IUs	755,077	776,173	776,163	841,500	831,263
Reported number of people treated	545,780	483,983	537,484	529,615	482,383
Programmatic coverage %	70.4%	62.4%	69.2%	62.9%	58.0%
Epidemiological coverage %	64.9%	57.5%	63.9%	62.9%	56.8%

Source: [28] PCT databank

#### MDA coverage survey

An independent coverage survey was conducted by lot quality assurance sampling in 2006 after the fifth MDA. It included all 4 subdivisions and 19 medical areas in the Northern region [29]. Each medical area was regarded as a “lot” except in large medical areas where nursing zone was considered a lot. A total of 27 lots was selected out of a possible 38 by population proportionate sampling. Villages were selected at random from a list and 13 households were selected in each village. The sample size was 2162 (1042 female, 1119 male, and one unknown); iTaukei Fijians were overrepresented due to incomplete listings for Indo-Fijian settlements. Persons of all ages in selected households were interviewed. Overall, 97.5% of eligible participants reported receiving the tablets and of those 96.8% reported taking the tablets. This is much higher than the program reported coverage. The reasons for this are not clear but have been attributed by the program to either social desirability bias in the survey, lack of recall, inaccurate registration or population overestimate, or a combination of these factors.

In program reports [27, 29], insufficient coverage in some divisions was attributed to incomplete drug distribution, lack of cooperation between village headmen and health workers, side effect fears, lack of transport of nurses to remote villages, inadequate promotion of the MDA program, size of the village, and the ability of the health workers to cover the area in the timeframe. Distribution of tablets at central points meant that it was unknown whether people actually took the tablets once they left the pick-up point. In contrast, there were high coverage reports in some divisions that were attributed to accessible transport for nurses, high level of community cooperation, awareness of public on MDA benefits and LF pathology, and active involvement of village headmen and health workers. Health workers distributing MDA were found to be the most important informational source about LF and MDA benefits. It was additionally noted that a shorter period of MDA distribution produced a higher coverage.

### Surveillance, monitoring, and evaluation of LF infection

#### Surveys

##### Baseline survey/A-survey

A summary of the baseline surveys done by subdivision in at least 45 sites between 1997 and 2001 is shown in Table S1. A total of 9350 people were tested. No Mf slides were done in these surveys. Detailed results by subdivision and village (where available) are shown in Table S1 in additional material.

Results by division and overall are given in Table 4. The baseline survey results showed that 16.6% of the 5983 people tested had a positive ICT result. However, the selection of the survey sites was not fully representative. Based on the results, the whole country was regarded as one implementation unit for nationwide MDA.

**Table 4:** LF prevalence in Fiji by division (positive ICT %)

Year(s)	Sampling method	Sample size	Division	No. sites	Antigen positive (ICT) %	Mf positive %
1997–2001 (A survey)	Convenience cluster	1443	Northern	3	14.8	
		307	Central	5*	42.0	
		2616	Eastern	24	20.2	
		1617	Western	13	7.7	
		5983	All Fiji	45	16.6	
2002 (pre-MDA survey)	Convenience cluster	0	Northern			
		1807	Central	25	13.9	8.1
		891	Eastern	15	23.9	6.8
		583	Western	8	2.1	0.2
		3281	All Fiji	48	14.5	6.3
2007 (C survey)	Stratified random cluster	894	Northern	8	3.0	
		1431	Central	12	15.4	
		3541	Eastern	37	11.1	
		879	Western	8	0.9	
		6745	All Fiji	65	9.5	1.4

*All on Beqa island, a suspected hotspot

Sources: PacELF databooks 2003 and 2006 [25, 26], The PacELF way [9], WHO Fiji office records

#### Mid-term/B-surveys

For monitoring the program, sentinel sites were selected in most subdivisions and sampled (B surveys) on two or three occasions, starting in 2002 (before the first MDA) and continuing in 2004 (after second MDA) and 2005 (after third MDA). Some additional sites were also sampled only once during this period. Full results of baseline and sentinel site surveys by subdivision are shown in Table S1, and results by site for those sites that were tested more than once in Table S2. Villages in Rewa subdivision (Central Division) were sampled extensively for a mosquito net project. The supplementary files demonstrate the extreme geographic heterogeneity observed in LF prevalence between village sites in Fiji.

The positive ICT percentage results of sites tested according to division and subdivision are shown in Table 5. Table 6 displays the positive Mf percentage results of populations tested according to division and subdivision. Included in these tables are the data for subdivisions which reported data for two or more years. Blank spaces in Tables 5 and 6 indicate no data were available for that year. The extreme range in prevalence and persistence in some areas to 2005 can be seen even at subdivision level, with Rotuma and Lau islands remaining at above 30% prevalence even after several rounds of MDA.

**Table 5:** Baseline and sentinel site surveys: antigen positive results by division and subdivision

Division	Subdivision	1997–2001			2002			2004			2005		
		No. sites	*N* tested	Ag pos %	No. sites	*N* tested	Ag pos %	No. sites	*N* tested	Ag pos %	No. sites	*N* tested	Ag pos %
Northern	Bua	1*	512	14.3									
	Cakaudrove	*1*	491	27.7							1	57	33.3
	Macuata	*1*	440	0.9									
Central	Naitasiri				4	248	26.2				1	97	26.8
	Rewa				21	1559	11.9	6	302	26.2	30	2619	6.3
	Serua/Namosi	*5*	307	42.0				4	233	27.0	5	410	14.9
Eastern	Kadavu	7	602	12.1							1	166	16.3
	Lau	12	1300	21.5	15	891	23.9				3	234	31.2
	Lomaiviti	*1*	551	11.6									
	Rotuma	*4*	163	67.5							4	176	35.8
Western	Ba	12	582	5.7	8	583	2.1	4	132	7.6	1	64	0.0
	Nadroga-Navosa	*1*	525	0.2									
	Ra	*1*	510	17.7									

*Lack of certainty on number of sites

**Table 6:** sentinel site survey Mf results by division and subdivision

Division	Subdivision	1997-2001			2002			2004			2005		
			Mf not done		No sites	N slides	Mf pos %	No. sites	N slides	Mf pos %	No sites	N slides	Mf pos %
**Northern**	Bua												
	Cakaudrove										1	57	3.5
	Macuata												
**Central**	Naitasiri				4	248	9.3				1	97	3.1
	Rewa				21	1559	7.9	4	288	6.9	30	2619	2.4
	Serua/Namosi							4	231	6.5	5	410	2.9
**Eastern**	Kadavu										1	166	3.6
	Lau				15	824	6.8				3	234	12.8
	Lomaiviti												
	Rotuma										4	176	10.8
**Western**	Ba				8	583	0.2		112	0.0	1	64	0.0
	Nadroga-Navosa												
	Ra												

#### Stop-MDA survey/C-survey

In 2007, the stop-MDA survey was completed via ICT testing in nationwide clusters (villages) which were selected at random from village lists. It included 65 village sites selected from the four divisions; there were up to 8 sites per division with the exception of Rotuma island in which 17 sites were sampled.

**Fig. 2:**
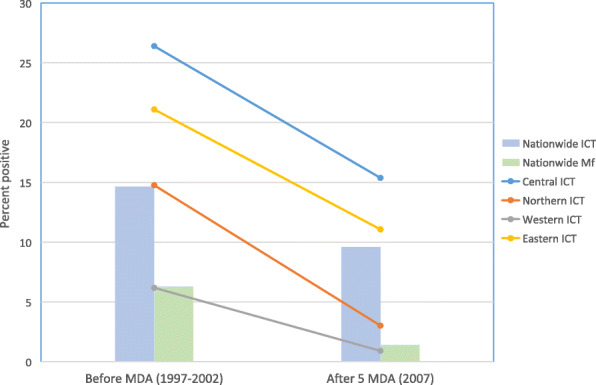
Prevalence of antigen and Mf positives before and after 5 rounds of MDA. Note: nationwide ICT prevalence before MDA is average of all antigen prevalence results in 1997–2001 and 2002. Northern Province was not surveyed in 2002. Mf prevalence data are only available for 2002 (excludes Northern Province) and 2007. See Table 4

Results are reported according to the four divisions within Fiji, which can be seen in Fig. 1, as well as reporting an overall national result. Results of the stop-MDA survey showed that on a national scale, 9.5% of persons tested were still positive, i.e., still well above the threshold of > 1% (Table 4). By division, the Northern, Eastern, and Central Divisions reported ICT prevalence > 1% (Table 4 and Fig. 2). The Western Division was the only region that “passed” the C survey with a 0.9% ICT prevalence, but the 95% CI was above 1%.

In Fig. 2, the antigen surveys done in 1997–2001 and in 2002 have been combined for the “pre MDA” baseline level, in order to increase the representativeness of the baseline in all divisions. The baseline Mf estimate is from 2002 only. Prevalence of positive ICT declined from the initial baseline survey result in all divisions. The decline was shallowest in Western Division which had the lowest baseline prevalence.

**Fig. 3:**
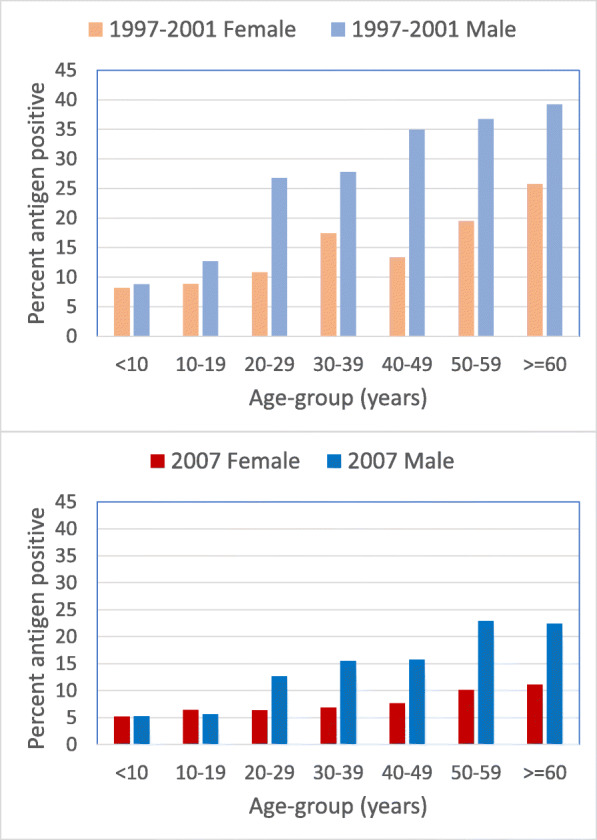
Age- and gender-specific prevalence of antigen in 1997–2001 A survey and 2007 C survey

Age-specific antigen prevalence nationwide also declined markedly between 1997–2001 and 2007 (Fig. 3), but was still above 20% in males over 50 years in 2007. Although the survey was not powered to estimate prevalence in particular age groups, nevertheless the presence of 5% of children under 10 (*N* = 1341 tested) who were antigen positive in 2007 suggests that transmission was still ongoing. Overall, the number of persons with known age and gender in the 2007 survey was 6540 out of 6745 tested.

As a result of the stop-MDA surveillance outcomes, Fiji was deemed not to be ready to terminate MDA and commence post-MDA surveillance, therefore continuing into its 6th round of MDA in 2008 after interruption of MDAs in 2007.

### Vector control

Vector control is also a recommended LF control strategy in Fiji as in all PacELF countries [9, 30], with the aim to reduce transmission between infected and uninfected persons. However, some species like *Ae.polynesiensis* may be difficult to control because of their extensive and unreachable natural habitats. Recommendations include larval habitat reduction, insecticide-impregnated bed nets and curtains, and use of repellent and mosquito coils [30]. A large study was done in 2003–2004 in Rewa district (near Suva in Central Division, see Fig. 1) to investigate whether use of long lasting insecticidal nets (LLIN) had any impact on LF prevalence, but the results did not find any significant effect [31]. This may not be surprising given that the main vectors are day-biting. Overall, prevention through vector control (e.g., removal of larval habitats) was apparently not a heavily emphasized part of the Fiji program (or at least there is no evidence of the extent to which it was done). Concurrently, mosquito control for dengue vectors (transmitted by *Ae aegypti* and *Ae albopictus* as well as *Ae polynesiensis* and the other LF vectors mentioned above) was also recommended and practiced in Fiji [32]. Methods included focal fogging with malathion, peri-domestic focal spraying with Aqua Resigen (bio-allethrin, permethrin, and piperonyl butoxide), larviciding with temephos, promotion of personal protection from bites, and larval habitat reduction (“clean-up campaigns”). These interventions were mainly done in urban areas, and their extent and timing is not known.

### Knowledge of filariasis and communication strategies

A knowledge, attitudes, and practices (KAP) feedback questionnaire was administered verbally to persons over 16 by trained health care workers in 2003, and allowed program staff to find weaknesses in knowledge and attitudes towards MDA and LF. KAP surveys were also carried out in 2005 and 2006 to investigate knowledge and inform strategies for increasing MDA participation.

The baseline KAP survey showed that the level of knowledge of filariasis was quite high, even before PacELF [9]. In 2003, two-thirds of the respondents said that mosquitoes transmitted LF, although 7.5% thought that “drinking dirty water” was the cause; responses such as “person to person contact” and “hygiene” were each given by less than 5% of the persons asked. More than half (57%) of people knew that taking medicine could prevent the spread of disease, and a further 32% mentioned mosquito control methods or nets for prevention [9].

Advocacy during the first two MDAs focused on printed materials. In the 2003 survey, most participants did not identify the printed materials as a source of information [27]. The program shifted more towards mass media (radio and TV) in all three vernaculars. In 2005, a KAP study was done in Rewa subdivision, and results showed increases in knowledge about the disease as well as awareness of the program [9]. For example, the proportion of people who knew that mosquitoes transmitted LF increased to 82%. Even though the proportion of people knowing that taking medicine could prevent LF remained relatively stable at 60%, a high proportion of 89% said they had taken the tablets at some time in the past. The main reason given for not taking the drugs was either missing or not knowing about the distribution [9].

In 2006, KAP questions were asked in conjunction with the coverage survey in Northern Division; 73.5% of 578 respondents knew that LF is transmitted by mosquitoes; 60% mentioned it was a cause of elephantiasis but only 20% considered it was a cause of hydrocoele [29]. The findings of the 2006 survey were used to develop and test an improved health promotion strategy with commercial media company input.

## Discussion

In Fiji, prevalence of LF was very high before the start of the PacELF MDA program, despite several previous attempts at widespread MDA [9]. A steep and successful decline in antigen prevalence was achieved between two nationwide surveys: the baseline survey (A Survey) in 1997 to 2002 (pre-MDA), and the results of the stop-MDA (C survey) in 2007. Antigen prevalence declined across all divisions and can likely be attributed to MDA and the supportive activities described in Table 1. Decline was observed both in overall and age-specific prevalence of antigen, and was more marked for Mf prevalence. However, the threshold for stopping MDA was not reached. Vector control (including that done for dengue control) may have contributed to the decline, but it is not possible to tell due to lack of information on this intervention.

Survey methodology improved over the time period described. Initial surveys in 1997–2002 (A surveys) used convenience sampling and were biased towards areas likely to have LF, in order to make sure that no endemic areas were missed. Participants in surveys consisted of those who chose to attend. The sentinel site B surveys were also purposively sampled and their sampling was not uniform, with a large number of sites sampled in Central Division, but by the 2007 stop-MDA C survey, clusters were selected randomly and generally in proportion to the population in each division. All surveys up to 2007 involved persons of all ages, which enabled the decline in age-specific prevalence in all ages to be demonstrated, although antigen prevalence over 20% persisted in males over 50 years.

There was large variation in prevalence between divisions, and decline in antigen prevalence was steepest in regions that started with higher prevalence. The results showed the importance of stratification for future program activities. Relying on a national average would mean that some areas would be undergoing unnecessary MDA, while other regions would stop MDA too early, allowing for a high-risk transmission environment to remain and lead to resurgence of LF. The sentinel site results show clearly the variation in prevalence and presence of hotspots within divisions and subdivisions as well.

Fiji was not able to reach the MDA coverage target (80% of the eligible population) according to the guidelines in place during 2002–2006 [9]. The current GPELF target of 65% of the total population [11] in all years was also not reached, according to the programmatic coverage reported. The nature of Fiji’s geography—multiple island populations and many remote areas—may have affected delivery of services and resources, including accuracy and completeness of the MDA registers, although there was also an indication from KAP surveys that coverage was not optimal in urban areas. The independent coverage survey in Northern Division in 2006 after the fifth MDA suggested that coverage may actually have been higher than programmatically reported, at least in some areas.

Findings of the repeated KAP surveys allowed staff to adjust promotion and education accordingly to maximize participation. Health promotion shifted to increase in urban settings as a result of the KAP feedback identifying a deficiency in this area. The renewed focus on addressing morbidity (both in terms of assessing the burden and addressing it) was also a major feature of the program after 2007 [33–35].

The extensive and well-sampled C survey showing the variation in prevalence by division enabled informed decisions to be taken on how to move forward. Despite the Western Division’s success in the stop-MDA survey, the decision was made by the WPRO regional program review group in 2008 [33] that all divisions in Fiji should continue with MDA, but that each division would be regarded as a separate implementation and evaluation unit with distinct timelines for post-MDA surveillance.

### Future of LF in Fiji

Fiji has had a challenging battle with LF, with many obstacles to overcome in order to effectively achieve the program goals, such as historically high prevalence, wide geographical population distribution, and funding. Looking back at program reports [33, 34], the standout challenge seemed to be MDA coverage and compliance. A study done in Central Division in Fiji in 2010 observed that participation in traditional village forums in Fiji was related to taking roles in community activities for MDA and associated with adherence to MDA regimen regardless of age, gender, and the educational attainment of the individual residents [36]. Looking forward, any future promotion endeavors required for MDA need to take the importance of community engagement into account.

Plans for the post-2007 period included dividing the country into 4 implementation/evaluation units by division [27, 32]; the Northern Division was later further subdivided further into two units. The WHO regional meeting in 2008 [33] recommended that MDA continue in Western, Northern, and Central Divisions, while Eastern would pursue a strategy of test and treat for positive cases. Directly observed treatment was introduced in Northern Division. A revised health communication strategy would be developed and implemented, and additional independent coverage surveys performed, with the next C survey planned in 2010. The WHO regional meeting in 2011 [34] noted that MDA was conducted in all 4 implementation units in 2008–2009, after which it stopped in Western Division. Central and Eastern Divisions continued MDA in 2010 and 2011; pre-TAS and TAS surveys were planned in Western Division in 2011 and in Central/Eastern Divisions in 2012. At the time of the 2011 meeting, MDA continued in Northern Division, while the test and treat program continued in remote island sites in Eastern Division. A renewed focus on identification of persons with morbidity and provision of services including hydrocoele surgery occurred after 2008 [34, 35].

Feedback regarding a national LF coordinator for Fiji suggested that implementation of someone in the position full time would have allowed for a more synchronized program between divisions. However, funding for this position was hard to secure and so there was a delay in establishing a representative in this role. There were issues with coordinating every island within the four divisions, and maintaining a consistent MDA availability across the entire division was a challenge as was the level of participation. Efforts intensified after 2007, but continuing transmission led to more MDA rounds in the face of continuing transmission, and the testing of triple drug MDA in the Eastern Division in 2018 [37].

There are several other Polynesian countries and territories with day-biting *Aedes* vectors (Niue, Cook Islands, Tonga, Wallis and Futuna) that have achieved validation of elimination of LF as a public health problem, although others are finding it difficult to reach the stop-MDA thresholds or are facing resurgence (Samoa, American Samoa, French Polynesia, Tuvalu) [10]. The reasons for success or difficulty are varied: some countries are small and relatively compact (Niue [38], Cook Islands [39]), have consistent and effective program managers despite being widely dispersed (Tonga [40]) or had relatively low prevalence at the start of PacELF (Wallis and Futuna [41]). Unlike some other countries in the region [42], the four successful countries/territories did not stop MDA until prevalence in all age groups was 1% or below, following PacELF guidelines [9]. Fiji’s large size and dispersed population together with lack of consistent program management made it unable to reach the needed MDA coverage levels between 2002 and 2006. Therefore, Fiji’s decision to continue MDA after 2007 with increased attention to monitoring, evaluation, communication, and morbidity was a wise one.

## Conclusions

During the years 1997 through 2007, Fiji dramatically reduced the overall prevalence of LF, particularly Mf prevalence, especially in Western and Northern Divisions. However, despite five rounds of MDA between 2002 and 2006 and large reduction in prevalence in all ages, the threshold for stopping MDA was not reached. The implementation of MDA and surveillance in conjunction with supportive activities in Fiji was challenging due to high starting prevalence, efficient mosquito vectors, extreme geographical spread of the population, and lack of consistent program management. The Fiji LF program has learned from persistence of transmission that has required adjustments and improvements to the elimination strategy over time, including multiple additional MDA rounds, application of better designed coverage and prevalence surveys, repeated revisions of implementation/evaluation units, improved communication strategies, attention to filariasis morbidity, and trials of delivery and surveillance approaches as it continues to pursue the goal of elimination of LF.

## Supplementary information

**Additional file 1.** Fiji LF Table S1 subdivision

**Additional file 2.** Fiji LF Table S2 Village

## Data Availability

Data presented within this paper belong to the Government of Fiji, and can be accessed with permission of Fiji’s Minister of Health.

## Abbreviations

LF: Lymphatic filariasis
DEC: Diethylcarbamazine citrate
GPELF: Global Programme to Eliminate Lymphatic Filariasis
ICT: Immunochromatographic test
MDA: Mass drug administration
Mf: Microfilaria or microfilariae
PacELF: Pacific Programme for Elimination of Lymphatic Filariasis
WHO: World Health Organization
SPC: Secretariat of the Pacific Community
DOT: Directly observed treatment
KAP: Knowledge, attitudes, and practice
